# Investigating the Causes of Non-adherence to Recommended Lifestyle Modifications Among Indian Patients With Newly Diagnosed Type 2 Diabetes Mellitus or Hypertension

**DOI:** 10.7759/cureus.78842

**Published:** 2025-02-11

**Authors:** Indu Saxena, Manoj Kumar, Babu L Kannoujia, Rajshri Aishwarya, Aniruddha Sen

**Affiliations:** 1 Biochemistry, All India Institute of Medical Sciences Gorakhpur, Gorakhpur, IND; 2 Physiology, Maharshi Vashishtha Autonomous State Medical College, Basti, IND; 3 General Medicine, Maharshi Vashishtha Autonomous State Medical College, Basti, IND; 4 Medicine, Uttar Pradesh University of Medical Sciences, Saifai, IND; 5 Biochemistry, Shri Gorakshnath Medical College Hospital and Research Center, Gorakhpur, IND

**Keywords:** : adherence, hypertension, lifestyle modification, non-adherence, types 2 diabetes

## Abstract

Background and objective

Lifestyle modification (related to diet and physical activity) is a key part of treatment for patients diagnosed with type 2 diabetes or hypertension. However, many patients are unable to follow the prescribed lifestyle modifications. This study aimed to investigate the causes of non-adherence in Indian adult patients with newly diagnosed type 2 diabetes mellitus (T2DM) or hypertension, who were advised lifestyle modifications along with pharmacotherapy. Problems encountered in observing adherence were recorded, and suggestions on how patient adherence may be improved were obtained after six months of treatment from patients and their family members using questionnaires and interviews.

Methods

A total of 80 patients (40 female) with newly diagnosed diabetes and/or hypertension, without any physical disability that would hinder exercise, were initially enrolled in the study. The patients and their families attended counseling sessions conducted at the hospital or at the patient’s residence at the time of treatment initiation. Twenty male patients dropped out within two months due to various reasons; their data were not included in this study. After six months of therapy, questionnaires related to lifestyle adherence were filled out by the patients. Patients and their relatives were interviewed regarding the problems associated with adherence and for suggestions on how adherence could be improved. Data obtained from 40 female and 20 male patients were used in the analysis.

Results

Adherence to lifestyle modifications was reported in all male patients who continued to participate in the study and 62.8% of female patients. All female patients with independent income reported good adherence. All patients reported their children's cooperation in helping them adhere to lifestyle changes. In the case of financially dependent female patients, adherence positively correlated with the presence of spouse and family members at the counseling session. A negative correlation was observed between the number of members in the household and the number of members who missed the counseling session. Regulation of diet and exercise was difficult in the case of female patients, especially those living in joint families.

Conclusions

Counseling the entire family is essential in the case of female patients to improve their adherence to lifestyle modifications. All children in the family who are old enough to comprehend dietary and physical activity changes should be included in the counseling sessions, which entail educating them about lifestyle diseases (obesity, T2DM, and hypertension).

## Introduction

The management of chronic, non-communicable diseases such as obesity, diabetes, and hypertension requires long-term treatment that involves adherence to recommended lifestyle and pharmacotherapy [1]. The World Health Organization has defined adherence as the extent to which a person’s behavior - taking medication, following a diet, and/or executing lifestyle changes (appropriate diet with increased physical activity) - corresponds with agreed recommendations from a healthcare provider. Adherence, unlike compliance, requires the patient’s agreement to the recommendations. The extent to which a patient does not follow a healthcare provider’s recommendations is termed non-adherence. Extreme non-adherence is termed attrition [1].

While adherence in developed countries is about 50%, the rates are lower in developing countries [1]. Poor adherence compromises the effectiveness of treatment, resulting in poor health outcomes and increased healthcare costs. The epidemiologic shift of disease burden from acute to chronic was recently disrupted by the COVID-19 pandemic. Individuals with chronic diseases like diabetes, hypertension, and cardiovascular diseases were more vulnerable to the infection and suffered more serious consequences of the infection [2], underlying the importance of management of these diseases.

Since the treatment/care required in chronic conditions predominantly relies on self-management on the part of the patient, it is important to investigate the factors that can increase the degree of adherence in these patients. Factors affecting adherence have been extensively investigated in developed countries [3,4], and these reportedly include sufficient knowledge about the condition, motivation by family and friends, and ease of executing lifestyle changes in terms of purchasing power. A developing country like India has a unique culture and societal norms, and hence the findings related to Western societies cannot be extrapolated to the Indian population.

This pilot study involves patients of Indian origin, with newly diagnosed diabetes mellitus, hypertension, or both, who had been advised lifestyle modification and pharmacotherapy. Counseling sessions were arranged for all patients and their family members to provide information regarding suitable lifestyle and its importance, and to provide tips on how to improve adherence. Factors affecting adherence and causes of non-adherence were investigated using questionnaires and one-to-one interviews with the patients and interested family members at the end of six months of treatment.

## Materials and methods

Study design, setting, and ethical approval

Both qualitative and quantitative data were collected and analyzed in this mixed-method research study. The study was conducted over one year (June 2023-June 2024), after obtaining ethical approval from the Institute Ethics Committee, Maharshi Vashishtha Autonomous State Medical College (MVASMC), Basti (reference no: IEC.ASMCB/ETHICS/004). Ethical clearance was also obtained at AIIMS Gorakhpur (IHEC/AIIMS-GKP/BMR/119/2023, dated March 23, 2023. Counseling services were provided to all patients requiring treatment; however, only adult patients with newly diagnosed diabetes and/or hypertension (who had not been practicing appropriate lifestyle modifications before their enrolment in this study) were selected for the study after obtaining their signed informed consent.

Hypertension was diagnosed according to the criteria defined by the American Heart Association: systolic blood pressure (SBP) ≥130 mmHg or diastolic blood pressure (DBP) ≥80 mmHg [5]. Diabetes mellitus was diagnosed in patients with HbA1c >6.5%, or fasting plasma glucose (FPG) higher than or equal to 126 mg/dL, or two-hour plasma glucose (2h PG) higher than or equal to 200 mg/dL during 75-gram oral glucose tolerance test (OGTT) [6]. Uniformity in condition was maintained to recruit patients with almost the same levels of therapy requirements. All patients were recommended lifestyle modification along with pharmacotherapy at the time of initiation to reduce cardiovascular risk. Lifestyle modification included weight reduction in patients who were overweight or obese; diet involving low fat, sodium, and sugar; physical activity; tobacco cessation; and moderation of alcohol consumption [7]. 

Inclusion and exclusion criteria

Inclusion Criteria

Patients with newly diagnosed type 2 diabetes mellitus (T2DM) who had been prescribed metformin monotherapy along with lifestyle changes, and patients with stage 1 or stage 2 hypertension who had been prescribed thiazide, angiotensin-converting enzyme (ACE) inhibitor, or both along with lifestyle changes, were eligible to participate. All patients belonged to families with a total monthly income exceeding Rs 10,000/- per family member. The socioeconomic status of the patients was considered important as it influenced the lifestyle of the patients.

Exclusion Criteria

Patients with chronic conditions other than the above, including conditions that could hamper physical activity, were excluded. Patients who had been prescribed any drug other than those mentioned above were also excluded from the study.

Data collection

Patients visiting the medicine OPD at MVASMC Basti or AIIMS Gorakhpur were recruited for the study. Initially, 80 patients (40 female) were recruited. However, 20 male patients failed to complete the study. One patient had an accident, three were transferred to other cities, and 13 patients chose to opt out of the study (without citing any reason) within the first two months. Three male patients did not appear at the end of six months of treatment for follow-up. Thus, data from 40 female and 20 male patients were included in the analysis.

Patients were categorized based on gender, financial status (independent and dependent), and type of family. The family was defined as a unit comprising members living together, sharing the same kitchen and financial resources. We grouped proton families (single-individual families) and electron families (no married couples) with nuclear families (single married couples with their children) for convenience. Similarly, atom families (nuclear family with any other family member(s) but no other married couple) and molecular families (exactly two married couples with or without unmarried people of any other generation) were grouped with joint families (two or more married couples of a single generation or three or more couples if multiple generations) [8].

Three female patients were unmarried, of whom two lived alone (proton families) and one lived with her parents. They were grouped under nuclear families. One female patient (widow) residing with her widowed mother, and one male patient (widower) residing with his two unmarried children (electron families) were also grouped under nuclear families. Details of patients are summarized in Table 1. Twenty-nine females (10 financially independent) and 12 male patients lived in joint families. One male patient was non-earning during the period of study.

**Table 1:** Details of the study participants

	Financially independent	Financially dependent	Nuclear family	Joint family
Female patients	19	21	11	29
Male patients	19	1	8	12

The age at the time of diagnosis of the disease was recorded among both female and male patients. The number of family members was counted as the number of people residing in the same house, sharing the same kitchen, facilities, and financial resources. Members who were residing separately (e.g., in hostels) were not counted even though they were financially dependent, as they could not physically be present in counseling sessions and did not influence the cooking pattern regularly. Members other than family were counted as those who were not part of the nuclear family. In the case of female patients residing in joint families, members other than family included the in-laws and their children or the married son and his family. In the case of male patients, the non-family members included the patient’s parents, his brothers and their families, and his unmarried sisters. All patients were asked to attend the counseling session at the time of initiation of treatment. Counseling sessions were organized at the hospital or at the patient’s residence at the time of treatment initiation.

Statistical analysis

Student’s t-test was used to compare the age at the time of diagnosis, number of children, number of household members, and number of members other than family between two groups (all females with all males, financially dependent and independent females, and members living in joint and nuclear families). The association between age at the time of diagnosis and the number of children, number of household members, and number of members other than family was analyzed using Pearson correlation.

## Results

Table 2 summarizes the details of age of onset, number of children, number of household members, and the number of members other than family.

**Table 2:** Various parameters among the study population SD: standard deviation

Parameter	All patients	
	Mean	SD
Age at the time of diagnosis, years	41.5	7.14
Number of children	1.95	1.09
Number of household members	6.2	3.07
Number of members other than family	2.53	2.71

The mean age at the time of diagnosis of condition (hypertension or type 2 diabetes mellitus) was 41.5 years (Table 2). The average age at diagnosis was significantly lower in females (39.4 years) compared to male patients (45.7 years) (Table 3). Male patients had a higher average number of children; however, this difference was not statistically significant. The number of household members and the number of members other than family were not significantly different between all male and all female patients.

**Table 3:** Comparison of age at TOD and family composition between female and male patients NoHM: number of household members; NoMOF: number of members other than family; SD: standard deviation; TOD: time of diagnosis

Parameter	Female patients		Male patients		t_Calculated_	P-value
	Mean	SD	Mean	SD		
Age at TOD, years	39.4	6.76	45.7	5.93	3.54	<0.0001
Number of children	1.85	1.2	2.15	0.79	1.02	0.322
NoHM	6.42	3.46	5.75	2.02	0.79	0.430
NoMOF	2.9	2.91	1.8	2.09	1.51	0.144

The age at diagnosis was significantly lower in financially dependent female patients (35.48 years) compared to financially independent female patients (43.74 years) (Table 4). No significant difference was observed in parity between the two groups. The number of household members and members other than family was higher in financially dependent female patients. These differences were highly significant.

**Table 4:** Comparison of age at TOD and family composition between financially dependent and independent female patients NoC: number of children; NoHM: number of household members; NoMOF: number of members other than family; SD: standard deviation; TOD: time of diagnosis

Parameter	Financially dependent		Financially independent		t_Calculated_	P-value
	Mean	SD	Mean	SD		
Age at TOD, years	35.48	4.59	43.74	6.09	5.88	<0.0001
NoC	2.10	1.27	1.58	1.04	1.58	0.119
NoHM	8.52	3.14	4.11	1.20	6.04	<0.0001
NoMOF	4.48	2.99	1.16	1.50	4.66	<0.0001

Table 5 compares the data of female patients residing in joint and nuclear families, irrespective of financial status. The difference in age at the time of diagnosis was of high magnitude and highly significant. Age at the time of diagnosis in patients living in joint families was 36.1 years, compared to 48.09 years in those living in nuclear families. Parity was significantly higher in women residing in joint families. A highly significant difference was seen in the number of household members between the two groups. The average number of household members was 7.83 for patients in joint families, compared to 2.73 for female patients in nuclear families.

**Table 5:** Comparison of age at TOD, parity, and NoHM between female patients living in joint and nuclear families JF: joint family; NF: nuclear family; NoHM: number of household members; SD: standard deviation; TOD: time of diagnosis

Parameter	JF (n=29)		NF (n=11)		t_Calculated_	P-value
	Mean	SD	Mean	SD		
Age at TOD, years	36.10	4.13	48.09	4.12	8.20	<0.0001
Parity	2.10	1.18	1.18	0.94	2.32	0.029
NoHM	7.83	2.97	2.73	2.24	5.15	<0.0001

Male patients living in joint families had a significantly lower age at diagnosis compared to those living in nuclear families (42.42 years compared to 50.63 years) (Table 6). The average number of children was 2.08 in joint families vs. 2.25 in nuclear families, with no significant difference between the two groups. As expected, the number of household members was significantly higher in joint families (6.83) compared to nuclear families (4.12).

**Table 6:** Comparison of age at TOD and family composition between male patients living in joint and nuclear families JF: joint family; NF: nuclear family; NoC: Number of children; NoHM: number of household members; SD: standard deviation; TOD: time of diagnosis

Parameter	JF (n=12)		NF (n=8)		t_Calculated_	P-value
	Mean	SD	Mean	SD		
Age at TOD	42.25	5.12	50.63	2.87	4.10	0.001
NoC	2.08	0.95	2.25	0.43	0.64	0.66
NoHM	6.83	1.91	4.12	5.99	0.16	0.002

The age at the time of diagnosis of medical condition in female and male patients showed a negative correlation with the number of children of the patient, the number of household members, and the number of members other than family (Table 7).

**Table 7:** Pearson correlation of age at TOD of medical condition with the number of children, household members, and members other than family (p<0.01) *P=0.048 TOD: time of diagnosis

Parameters	All patients (n=60)	Female patients (n=40)	Male patients (n=20)
No. of children	-0.1063	-0.1876*	-0.15
No. of household members	-0.6452	-0.6718	-0.6856
No. of members other than family	-0.6244	-0.6377	-0.5335

Surprisingly, in the case of female patients who were financially dependent or were living in joint families, the age at the time of diagnosis positively correlated with parity (p<0.05) (Table 8). A strong positive correlation was observed between age at diagnosis and financial independence in the case of female patients residing in joint families.

**Table 8:** Pearson correlation of age at TOD with parity, NoHM, and NoMOF in between female patients of different groups (p<0.05) FIND: financially independent; NA: not applicable; NoHM: number of household members; NoMOF: number of members other than family (NoMOF=0 in nuclear families) TOD: time of diagnosis

Parameter	All female patients (n=40)	Financially independent patients (n=19)	Financially dependent (n=19)	In joint families (n=29)	In nuclear families (n=11)
Parity	-0.1876	-0.3660	0.2654	0.2656	-0.2399
NoHM	-0.6718	-0.5476	-0.0214	-0.3497	-0.3055
NoMOF	-0.6377	-0.3712	-0.0789	-0.3706	NA
FIND	0.6103	NA	NA	0.6493	0.0103

Spouses and/or family members of 31 female patients and 19 male patients attended the counseling session. A total of 35 female patients had spouses living with them, of whom 15 attended the counseling session with their wives (Table 9). Thirty-three families of female patients had children living with them. One or more children of 13 families attended the counseling session. Twenty-nine female patients were living in joint families, of which 26 had a father- or mother-in-law, or both. Parent(s)-in-law of only four female patients attended the session, even if the session was conducted at their residence (in case of large families). Parents-in-law attending the counseling session had the same medical condition (diabetes or hypertension) as their daughter-in-law. Other in-laws (brother- or sister-in-law, or both, with/without their children) were present in 19 families. Only nine female patients were accompanied by their sister-in-law. More husbands accompanied their financially independent wives (Table 9).

**Table 9:** Details of household members participating in the counseling session in the case of female patients FDEP: financially dependent female patients; FIND: financially independent female patients; JF: joint family; NF: nuclear family; OIL: household members related by marriage, other than parents-in-law; PIL: parent-in-law (Note: numbers of in-laws = 0 in nuclear families)

Relationship with patient	Total no. of patients with this relation	Total no. of patients with the relative attending counseling session		Relative attending from				
				JF (n=29)	NF (n=11)	FIND (n=14)	FDEP (n=21)	
		Number	Percent					
Husband	35	15	42.86	10	5	9	6	
Child	33	13	39.39	9	4	5	8	
PIL	26	4	15.38	4	0	2	2	
OIL	19	9	47.37	9	0	1	8	
Mother	2	2	100	0	2	2	0	
Father	1	1	100	0	1	1	0	

Nineteen male patients had their spouses living with them, and they attended the counseling session (Table 10). All male patients had their children living with them. One or more children of 13 male patients attended the counseling session. Ten male patients with joint families had their parent(s) living with them. All living parents attended the counseling, even if the session was held at the hospital. Five male patients had siblings living with them. Sibling(s) in four families attended the counseling.

**Table 10:** Details of household members participating in the counseling session in the case of male patients (n=20) JF: joint family; NF: nuclear family (Note: numbers of parent(s) or siblings = 0 in nuclear families)

Relationship with patient	Total no. of patients with this relation	Total no. of patients with the relative attending counseling session		Relative attending from		
				JF (n=12)	NF (n=8)	
		Number	Percent			
Wife	19	19	100	12	7	
Child	20	13	65	6	7	
Parent	10	10	100	10	0	
Sibling	5	4	80	4	0	

All male patients and all financially independent female patients reported adherence to lifestyle modifications. In the case of female patients, self-reported adherence negatively correlated with parity (non-significant) and numbers of household members and members other than family (significant) (Table 11). Since the family size was highly variable, the correlation of adherence with the number of family members not attending counseling sessions was checked, and a strong negative correlation was observed. A highly significant positive correlation was observed between adherence and the presence of a spouse in counseling sessions and financial independence.

**Table 11:** Correlation of self-reported adherence to lifestyle changes with family composition in the case of female patients NA: not applicable; NoHM: number of household members; NoMOF: number of members other than family (NoMOF = 0 in nuclear families) Note: financial independence is considered 0 in financially dependent females

Parameter	All female patients (n=40)	Financially dependent (n=19)	In joint families (n=29)	In nuclear families (n=11)
Parity	-0.0108*	0.2654	0.2591	-0.6143
NoHM	-0.3829	-0.0214*	-0.1721	-0.6329
NoMOF	-0.3633	-0.0789	-0.2042	NA
Presence of spouse in counseling session	0.5164	0.3162	-0.4989	0.1789
Family members not attending counseling	-0.5525	-0.3347	-0.4306	-0.6708
Financial independence	0.7368	NA	0.7009	0.6708

Table 12 summarizes the problems encountered in adherence to lifestyle modifications, by both female and male patients. One problem encountered by almost all patients was resisting temptation (of eating unhealthy food or avoiding exercise), which is not included in the table.

**Table 12:** Summary of problems encountered in adherence by different patient groups

Adherence		Family type	Problems encountered
Yes	Males (n=20)	Joint family (12)	Increased expense related to different types of meals; lack of time for exercise; difficulty in abstaining from tobacco and/or alcohol
		Nuclear family (8)	
Yes	Financially independent females (n=19)	Joint family (10)	Increased expense related to different types of meals; lack of time and space for exercise; lack of time to prepare different meals; increased frequency of purchasing convenience food for other family members
		Nuclear family (9)	Family members frequently become bored with "healthy food"
No	Financially dependent females (n=21)	Joint family (19)	Increased expense related to different types of meals; joint family members objecting to "special meals" for non-earning housewives; family members refusing to eat healthy food due to eating habits; lack of time to prepare different meals; family members object to wastage of unhealthy leftovers; expect the housewife to finish unhealthy leftovers Lack of time, space, and privacy to exercise; lack of time and money to join a gym for regular exercise
		Nuclear family (2)	Family members frequently become bored with "healthy food"

## Discussion

A total of 60 patients (40 females) completed this study (Table 1). Nineteen female (almost half the total number) and 19 male patients were earning. Of note, 29 female and 12 male patients lived in joint families. More participants belonged to joint families. The age at the time of diagnosis and the composition of patients’ families are summarized in Table 2. While the mean age at the time of diagnosis of the medical condition was 41.5 years for all patients (Table 2), a comparison between female and male patients showed a highly significant difference (Table 3). The mean age at the time of diagnosis was 39.4 years compared to 45.7 years in the case of male patients. Other parameters like number of children, number of family members, and number of members other than family did not show any significant difference between the two groups (Table 3).

The age of the patient at the time of diagnosis showed significant variation between financially independent and dependent females (Table 4). The mean age at the time of diagnosis was 35.5 years in the case of financially dependent female patients, while it was 43.7 years in the case of financially independent females. Although the average number of children was higher in the financially dependent group, this difference in parity was not significant. Highly significant differences were observed in the number of household members and members other than family between the two groups.

Female patients living in joint families showed similarity with the financially dependent group of female patients (Table 5). The mean age of diagnosis was significantly lower in the case of female patients living in joint families (36.1 years) compared to those living in nuclear families (48.1 years). A small but significant difference was observed in parity between the two groups. This is because three females included in nuclear families were unmarried, and without children, and one patient, a widow living with her mother, had a child who was not living with her. Number of household members was much higher in case of female patients living in joint families, as was expected. A comparison of age at the time of diagnosis between male patients living in joint and nuclear families showed a similar pattern (Table 6). The mean age of diagnosis was 42.4 years in patients residing in joint families, compared to 50.6 years in male patients residing in nuclear families. While the difference in the number of children was not significant between the groups, the average number of household members was significantly higher in the case of male patients living in joint families.

The data presented in Tables 4-6 suggest that a higher number of household members, and a higher number of members other than the family may be associated with increased chronic stress in both female and male patients, resulting in earlier onset and diagnosis of disease in these patients. In the case of female patients, financial dependence is an additional factor associated with earlier age of diagnosis. This is supported by the strong, significant negative correlation observed between the age at the time of diagnosis and the number of household members and the number of members other than family in both female and male patients (Table 7). Chronic stress is a risk factor for obesity, hypertension, and diabetes [9-11]. A higher number of household members could lead to chronic stress in families with unequal distribution of authority. A weak negative correlation (p>0.05) was observed between age at the time of diagnosis and the number of children, indicating that a larger number of children was associated with a younger age of onset of the medical condition.

Family dynamics (patterns of interactions among family members living together) influence the development and well-being of an individual via psychosocial, behavioral, and physiological pathways [12]. The health outcomes of unhealthy family dynamics have been established in children. Adverse childhood experiences, or ACEs, are known to increase the risk of heart, lung, or liver disease, depression, and anxiety [13]. In the case of adults, immunity decreases with age. The stress of poor marital quality may accelerate the aging of the immune system, especially in women [14]. Aging increases the vulnerability to stress, resulting in exacerbation of chronic conditions [15].

A strong positive correlation between age at diagnosis and financial independence was observed in women, especially those residing in joint families (Table 8). Financially dependent or non-earning women are probably more vulnerable to family dynamics and possibly experience greater stress, especially in joint families. Most of the literature related to the influence of family dynamics on women is limited to domestic violence and maternal health. The ability of women to make decisions about household matters influences the likelihood of utilizing healthcare. In South Asia, women having greater agency or authority to make decisions are more likely to use maternal healthcare [16]. Yeom and Lee [17] have shown that the effect of family function is significant in women, but not in men.

Since we did not investigate the stress level in the patients we cannot definitely comment on the cause of lower age at the time of diagnosis in female patients, especially those who were financially dependent. However, the number of family members attending the counseling sessions suggests the involvement of family dynamics in the lower age of diagnosis in women (Table 9). In the case of female patients, 35 women had spouses living with them, of whom only 15 (42.8%) attended the counseling session. Wives of all 19 married men attended the session (Table 10), suggesting greater importance of the husband’s health in comparison with the wife’s health. Husbands, children, and other relatives of financially independent women attended the counseling sessions in greater numbers (Table 9), indicating the importance of the earning status of the woman. Apparently, the value of unpaid household work still lacks recognition in many families.

Data in Tables 9-10 compare the family members attending counseling in the case of female and male patients. Of note, 33 Women had their children living with them. Children from 13 families (39%) attended counseling, compared to 65% in the case of men. Both female and male patients reported that their children had supported the change in their lifestyle by adopting a similar lifestyle, encouraging walks, and asking other family members for cooperation. All parents living with their sons attended the session, and showed their concern regarding their son’s health (Table 10), compared to 15% attendance by parents-in-law for women (Table 9). Eleven counseling sessions were held at the patient’s home due to the large family size; the parents-in-law were unable to attend. Parents-in-law who attended the counseling had the same medical condition as the patient, suggesting the attendance was more in self-interest rather than in concern for the daughter-in-law’s health. Of note, 80% of siblings of male patients attended the counseling, compared to 47% of other in-laws (brothers- or sisters-in-law) in the case of female patients. This shows an element of discrimination against the women in the family.

Self-reported adherence positively correlated with parity in the case of financially dependent women and in women living in joint families (Table 11). A strong positive correlation was observed between adherence and the presence of a spouse at the counseling session. This suggests that the presence of the husband influenced family dynamics in favor of the woman patient. Adherence negatively correlated with numbers of household members and members other than family; the reason is apparent from the problems encountered by the patients (Table 12). A strong negative correlation was seen between adherence and the number of household members not attending the counseling. No questions were asked regarding reasons for the absence of family members, but interviews with the female patients and the difficulties they encountered in adherence to the suggested lifestyle modifications signified a lack of good family relationships.

Lack of information was not a cause of non-adherence. Almost all patients reported difficulty in adhering to the recommended diet and exercise or abstaining from tobacco and/or alcohol (seven male patients). Physical exercises that did not require gadgets were taught during counseling so that all patients would be able to exercise. Healthy food comprising fruits and vegetables is relatively more expensive than convenience food. Purchasing healthy food for all family members was often difficult in joint families with a greater number of family members. Budget problem was often encountered by both female and male patients (Table 12), except by unmarried women.

Female patients in India have been reported to show less adherence to lifestyle modifications as compared to male patients [18]. In this study also, most of the problems were encountered by women living in joint family households, where family members refused to cooperate in matters of diet and exercise. Lack of family support in adherence to diet and physical activity was apparent in the case of many female patients. Females who were overweight or obese reported the blaming attitude of family members. One joint family split at the end of the study period due to this reason, underlining the importance of healthy cooperation by household members. Two other joint families (one involving a male patient) reported conflicts due to a lack of space and privacy for growing children and announced their plans to split into two or more families. Twenty-three Female patients needed our assurance that their medical condition and obesity were not their fault, or that their weight problem had not been brought upon them by themselves. Consequently, we included a brief discussion on the common causes of obesity [9] in the second counseling session held after six months.

Adherence to an appropriate lifestyle requires knowledge, motivation, and support from family members. Support from spouses is very important to female patients, especially those who are financially dependent and living in joint families. Children should be included in counseling sessions if they are old enough to understand, as they can advocate for their mother in matters of cooperation from family members.

Limitations of the study

This study has a few limitations, primarily its small sample size, especially in the case of male patients. The 50% attrition rate in the case of male patients recruited in the study was unexpected and may have affected our results, reflecting a false adherence rate in the case of male patients. We recommend larger studies to establish the causes of non-adherence in female and male patients in the different regions of India, as social and cultural values may vary from region to region.

There is a need to assess chronic stress levels in adult patients diagnosed with obesity and obesity-related diseases. There is also a need to provide counseling sessions to entire families regarding the importance of healthy lifestyles and family support in the management of non-communicable diseases like diabetes and hypertension.

## Conclusions

Adherence to the recommended lifestyle is an essential part of therapy in cases of chronic non-communicable diseases like diabetes and hypertension. Adherence requires knowledge as well as the support of family members, which can be enhanced by organizing counseling sessions for the entire family. Children of both female and male patients can help in achieving healthy lifestyles. Persons addicted to alcohol/tobacco face difficulty in adhering to dietary modifications, and often require pharmacotherapy. Dietary modification may incur additional expenses, especially in the case of large households. Increasing physical activity via exercise or other activities is difficult for female patients living in small homes in neighborhoods without parks. Adherence in female patients can be improved by enlisting the support of the husband and family members. This can be achieved by encouraging the attendance of family members in counseling sessions.
